# Ultrasonographic Evaluation of Masseter Muscle Thickness and Fractal Dimension in Bruxist and Healthy Individuals

**DOI:** 10.3390/diagnostics16213648

**Published:** 2026-11-04

**Authors:** Esin Akol Görgün, Ayşegül Öndeş

**Affiliations:** Department of Oral and Maxillofacial Radiology, Faculty of Dentistry, Adıyaman University, Altınşehir Mah. 3005 Sok. No: 13, 02040 Adıyaman, Türkiye; aondes@adiyaman.edu.tr

**Keywords:** bruxism, masseter muscle, ultrasonography, fractal analysis, muscle thickness

## Abstract

**Background/Objectives**: This study aimed to determine whether masseter muscle thickness and ultrasound-derived fractal dimension (FD) differ between patients with bruxism and healthy controls. **Methods**: Thirty patients with clinically assessed bruxism and 30 healthy volunteers (15 female, 15 male each) were included. Bruxism required a history of tooth grinding or clenching for at least one year plus at least one of three additional findings (tooth wear, masseter tenderness on palpation, morning jaw pain or headache); controls had none of these findings. Bilateral B-mode scans were acquired at rest and during maximum clenching under standardized settings. Thickness was measured with calipers, and FD was calculated by box-counting in ImageJ (version 1.54f) from three 100 × 100-pixel regions of interest per image. Intraobserver reliability was excellent for thickness (ICC = 0.967–0.980) and good for FD (ICC = 0.850–0.929). **Results**: Thickness was greater in bruxism patients on both sides and in both conditions (*p* < 0.001; bilateral mean at rest 12.95 ± 1.47 vs. 9.72 ± 1.78 mm). Bilateral mean FD was higher in bruxism patients during clenching (1.202 ± 0.027 vs. 1.182 ± 0.028; *p* = 0.008), and this difference remained significant after Holm correction across six FD comparisons. The resting difference (1.188 ± 0.026 vs. 1.171 ± 0.031; *p* = 0.029) was not robust to multiplicity correction or to exclusion of two controls whose regions of interest extended beyond the muscle. **Conclusions**: Clinically assessed bruxism was associated with greater masseter thickness in this sample. Bilateral mean FD was modestly higher during clenching; the resting FD difference was not robust. The reproducibility and clinical relevance of these texture measures require further study.

## 1. Introduction

Bruxism involves rhythmic or non-rhythmic masticatory muscle activity during sleep or wakefulness that results in tooth clenching and jaw muscle contractions [1]. Sleep bruxism affects roughly 10% of adults; the awake form is reported by about 30%, with women showing higher prevalence rates in both categories [2,3]. Tooth wear, cervical dentin hypersensitivity, tooth sensitivity, restoration fractures, muscle pain, TMJ pain, and TMJ sounds are among the clinical consequences [4].

Alcohol, tobacco, caffeine, and various medications have been implicated in the etiology [4,5,6], alongside poor nutrition, sleep-disordered breathing, lifestyle factors, psychological stress, anxiety, depression, and genetic susceptibility [7,8,9,10,11]. Because the condition is multifactorial—biological, external, and psychosocial components all contribute—management typically requires a multidisciplinary approach that may encompass pharmacotherapy, occlusal splints, physical therapy, and behavioral or psychosocial interventions [12,13].

Diagnosis relies on patient-reported information, clinical examination findings, and polysomnographic data [14]. The Standardised Tool for the Assessment of Bruxism (STAB), developed in recent years, integrates medical history, clinical signs, and supplementary investigations into a structured assessment framework that has become central to accurate bruxism classification [15,16].

Treatment decisions are driven by the impact on oral and dental tissues, temporomandibular joint function, and associated neurological or sleep-related symptoms including headache and muscle tenderness [17,18]. The goal is to limit damage and relieve symptoms.

Bone-muscle mechanical and biochemical interactions may lead to bone remodeling in bruxism patients. Excessive loading not only causes bone remodeling but also affects muscle mass through the release of growth-regulating factors [19]. Muscle tissue can be assessed with imaging modalities such as ultrasonography (USG), magnetic resonance imaging (MRI), and computed tomography (CT), whereas electromyography (EMG) provides a separate, electrophysiological assessment of muscle activity. Due to its dynamic and real-time imaging capability, high resolution, ease of application, low cost, and reproducibility, USG has been reported as a reliable method for the in vivo assessment of muscles [20,21,22].

Some subtle details in any image may not be noticed by the human eye. Such subtle details in a medical image may conceal potential pathological lesions, degenerations, inflammation, and even tumors. Fractal geometry provides a mathematical framework for describing complex, self-similar biological structures; fractal dimension (FD) serves as a quantitative, image-derived descriptor of spatial pattern complexity and irregularity [23]. In biological systems, alterations in fractal properties reflect underlying changes in tissue organization [24]. The subtle geometry of tissue medical images obtained through USG can be characterized by complex parameters such as fractal analysis [24]. Studies on fractal analysis in dentistry exist using panoramic, periapical, bitewing radiography, cone-beam computed tomography (CT), micro-CT, sialography, and USG [25]. In the dental literature, FD has been explored in masseter ultrasonography in the context of TMJ disorders [26] and probable bruxism [27], following botulinum toxin injection [28], and in mandibular bone radiographic analysis in bruxism patients [29]. However, there are very few studies on fractal analysis of USG images of the masseter muscle [26,27,28]. Beyond dental radiology, FD has also been applied to soft-tissue MRI applications such as cardiac muscle texture analysis [30] and brain tumor characterization [31].

The present study imaged bilateral masseter muscles in bruxism patients and healthy controls using USG, then compared muscle thickness and FD values between the two groups.

## 2. Materials and Methods

### 2.1. Study Group

The study was carried out at the Faculty of Dentistry, Adıyaman University, under approval from the Adıyaman University Ethics Committee (Decision No: 2026/6-8; 17 September 2026). Participants were recruited, and their ultrasonographic images were acquired, between 18 and 28 September 2026. Bruxism patients were recruited consecutively from individuals presenting to the Department of Oral and Maxillofacial Radiology. Control participants were recruited from individuals attending the same faculty for other dental procedures and from healthy volunteers with no bruxism-related complaints. The equal distribution of women and men (15 per group) was planned in advance to enable sex-stratified comparisons. Thirty bruxism patients (15 female, 15 male; mean age 28.9 ± 8.7 years) and 30 healthy controls (15 female, 15 male; mean age 29.5 ± 8.2 years) were enrolled. Written informed consent was obtained from every participant.

Bruxism diagnosis was based on a structured clinical interview and examination consistent with the Standardised Tool for the Assessment of Bruxism (STAB) framework [15,16]. Participants were required to meet at least two of four criteria, one of which was mandatory: (1) a patient-reported history of tooth grinding or clenching for at least one year (mandatory criterion); and at least one of the following additional criteria: (2) clinically observed tooth wear consistent with bruxism; (3) masseter muscle pain or tenderness on palpation; (4) patient-reported morning jaw pain or headache. The one-year requirement was applied at recruitment; patients reporting grinding or clenching for less than one year were not included. Criteria (1) and (4) were assessed by history and criteria (2) and (3) by clinical examination. In the bruxism group, all 30 participants met criterion (1); tooth wear was present in 14 (46.7%), masseter pain or tenderness on palpation in 18 (60.0%), and morning jaw pain or headache in 23 (76.7%). This author-defined clinical rule was informed by the STAB framework but does not represent the application of a validated diagnostic algorithm. Control participants underwent the same structured interview and clinical examination; individuals were included in the control group only if none of these findings were present. Sleep and awake bruxism were queried during the clinical interview, but the subtype was not systematically recorded; subtype-specific data are therefore not available, and the study addresses clinically assessed bruxism as a whole.

Exclusion criteria were: neuromuscular disorders, rheumatoid arthritis, fibromyalgia, uncontrolled diabetes mellitus, or thyroid dysfunction; use of muscle relaxants, botulinum toxin, or corticosteroids; temporomandibular joint disorders—participants with joint-related TMD findings (joint sounds, restricted mouth opening, or joint pain on palpation) were excluded from both groups; missing posterior teeth; prior masseter-region surgery; and pregnancy. Masseter muscle pain or tenderness, a muscle-origin finding distinct from joint pathology, was used as one of the bruxism inclusion criteria and was not an exclusion criterion. Joint-related findings led to exclusion, while muscle-related findings contributed to bruxism classification.

### 2.2. Ultrasonographic Evaluation

Clinical assessment and group allocation were performed by one examiner, who referred participants for imaging. All ultrasound examinations and FD analyses were performed by a second examiner, an experienced clinician who was not informed of the participants’ group assignment (bruxism vs. control) during image acquisition or analysis. Scans were performed using a GE Versana Premier device (GE Healthcare, Chicago, IL, USA) with a 12L probe. The following device settings were standardized and maintained constant throughout the study: frequency 10.0 MHz, gain 51 dB, depth 4.0 cm, dynamic range 66 dB, spatial averaging 3/4, gray map F/0, Whizz off (Small Parts preset). The probe was held perpendicular to the skin surface with minimal and consistent pressure using a generous amount of coupling gel to avoid tissue compression. Participants sat upright with the Frankfort horizontal plane parallel to the ground. Both right and left masseter muscles were examined.

**Figure 1:**
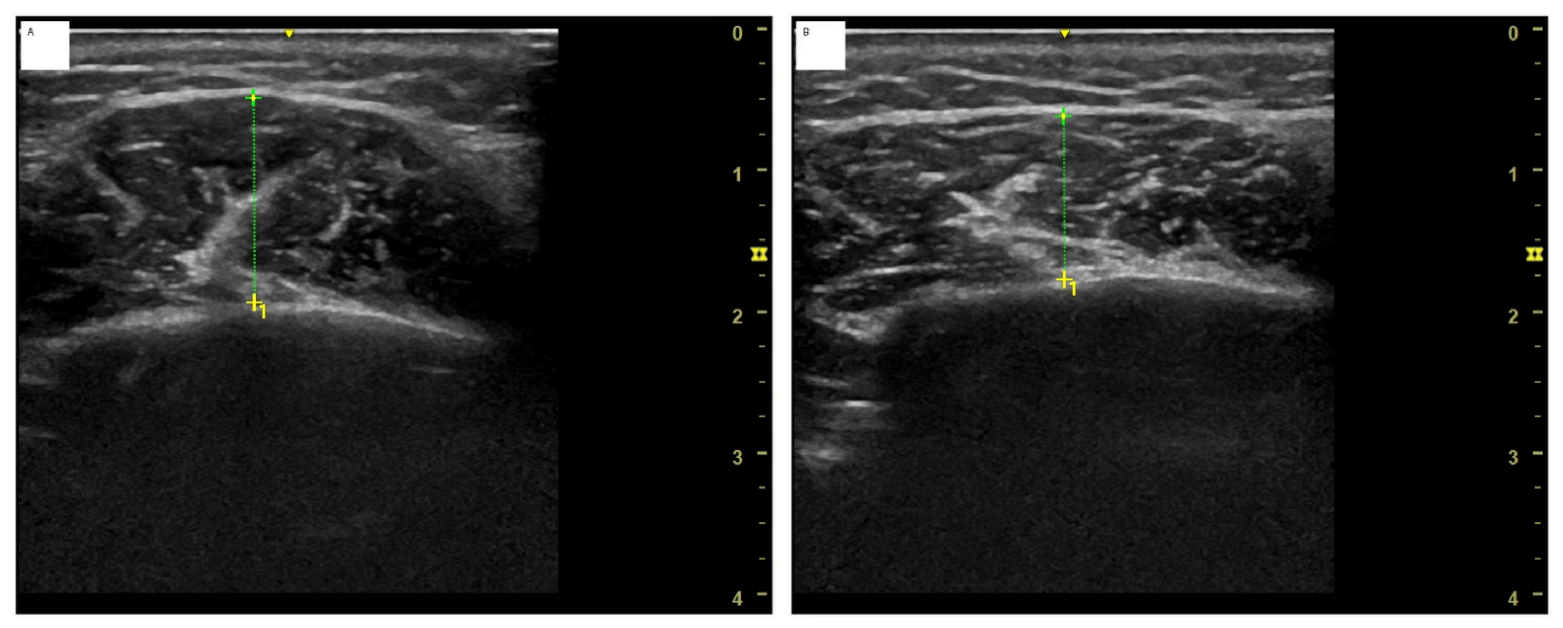
B-mode ultrasonographic images of the masseter muscle during maximum voluntary clenching (**A**) and at rest (**B**). The green dotted line indicates the thickness measurement taken perpendicular to the surface of the mandibular ramus at the midpoint between the zygomatic arch and the inferior border of the mandible. The depth scale (cm) is displayed on the right side of each image; image depth was 4.0 cm.

B-mode thickness measurements were taken at rest and during maximum voluntary clenching (Figure 1). For the resting condition, participants were instructed to part their lips slightly with teeth not in contact and the tongue resting passively on the floor of the mouth. For the clenching condition, participants were instructed to bite down as hard as possible on their own posterior teeth for five seconds, during which the image was acquired; a standardized verbal instruction was used for all participants. The probe was placed horizontally, parallel to the inferior border of the mandible and therefore transverse (perpendicular) to the masseter muscle fibers, at the midpoint between the zygomatic arch and the inferior mandibular border. For each side and condition, two images were acquired with a 30-s rest interval between them. Muscle thickness was measured once per image using the CALIPER function, perpendicular to the surface of the mandibular ramus, between the superficial and deep fascial boundaries, with calipers placed at the muscle–fascia interfaces [32]. The mean of the two measurements was used as the final participant-level value, as illustrated in Figure 1.

### 2.3. Fractal Analysis

**Figure 2:**
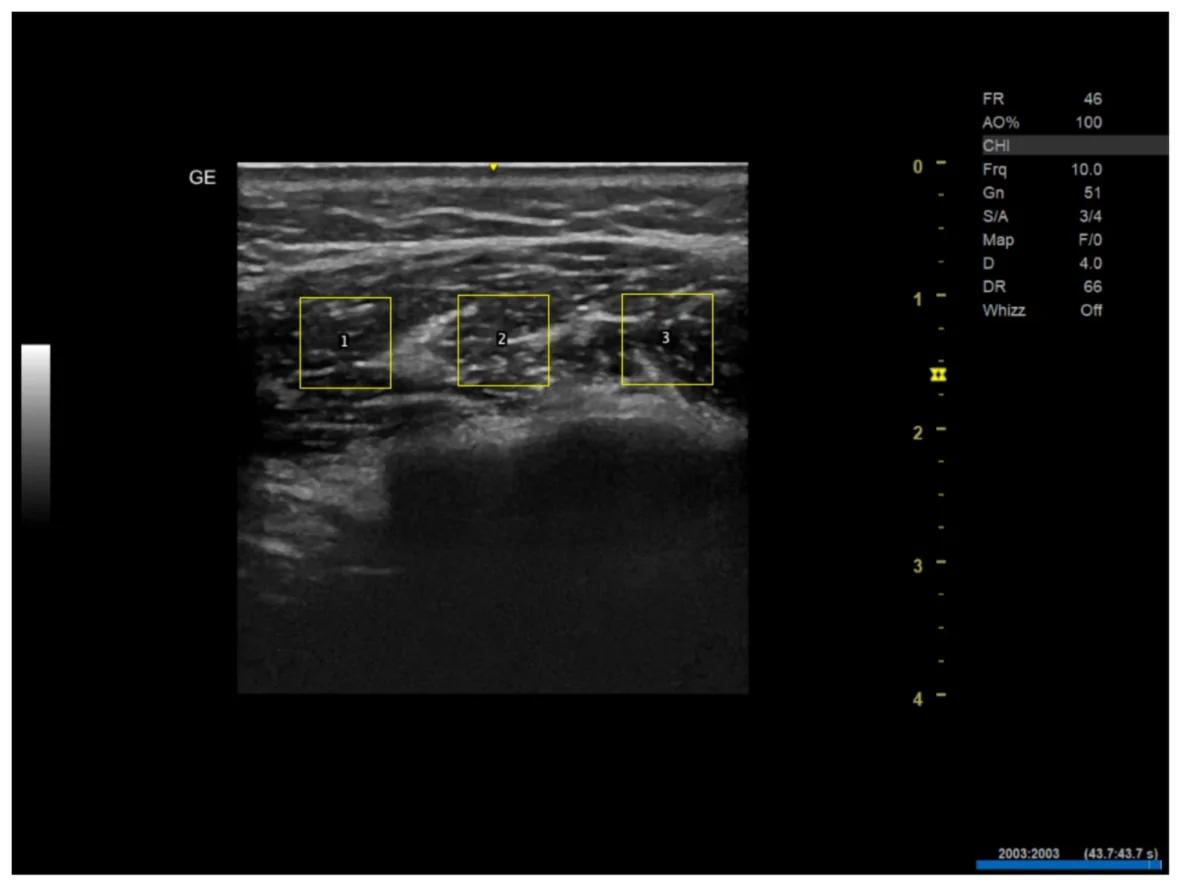
B-mode ultrasonographic image of the masseter muscle at rest showing the three regions of interest (ROI) selected for fractal analysis at the anterior (1), middle (2), and posterior (3) portions of the muscle. The depth scale (cm) is displayed on the right side of the image; image depth was 4.0 cm.

FD values were extracted from B-mode images using ImageJ version 1.54f (NIH, Bethesda, MD, USA) and its built-in Fractal Box Count function. Images were exported from the ultrasound device as standard (compressed) JPEG files at the native pixel resolution (depth 4.0 cm; pixel spacing approximately 0.068 mm, determined in ImageJ by measuring the 4-cm depth scale of the exported image with the straight-line tool; the ROI side length was identical in the horizontal and vertical directions, indicating square pixels, so each 100 × 100-pixel ROI represents approximately 6.8 × 6.8 mm of tissue; because all images were acquired with the same depth setting and device settings and exported in the same way, this calibration applied to all images) without resizing. In each image, three 100 × 100-pixel ROIs were placed within the muscle, between the superficial and deep fascial boundaries, at the anterior, middle, and posterior one-thirds of the visible muscle belly, identified at the midpoint between the zygomatic arch and the inferior border of the mandible. The same ROI size was used in all images. In the resting images of two control participants, the muscle was thinner than the ROI height (6.8 mm), so all ROIs in these images (18 ROIs in six images) necessarily extended beyond the muscle and included adjacent non-muscle tissue. In all other images, including all clenching images, the ROIs lay entirely within the muscle; this was verified by re-inspecting the images of the participants with the thinnest muscles; in all other participants, the measured muscle thickness exceeded the ROI height by more than 2 mm in every image. The numbers and ROI outlines shown in Figure 2 were added post-hoc for illustration only and were not included in the analyzed image data.

**Figure 3:**
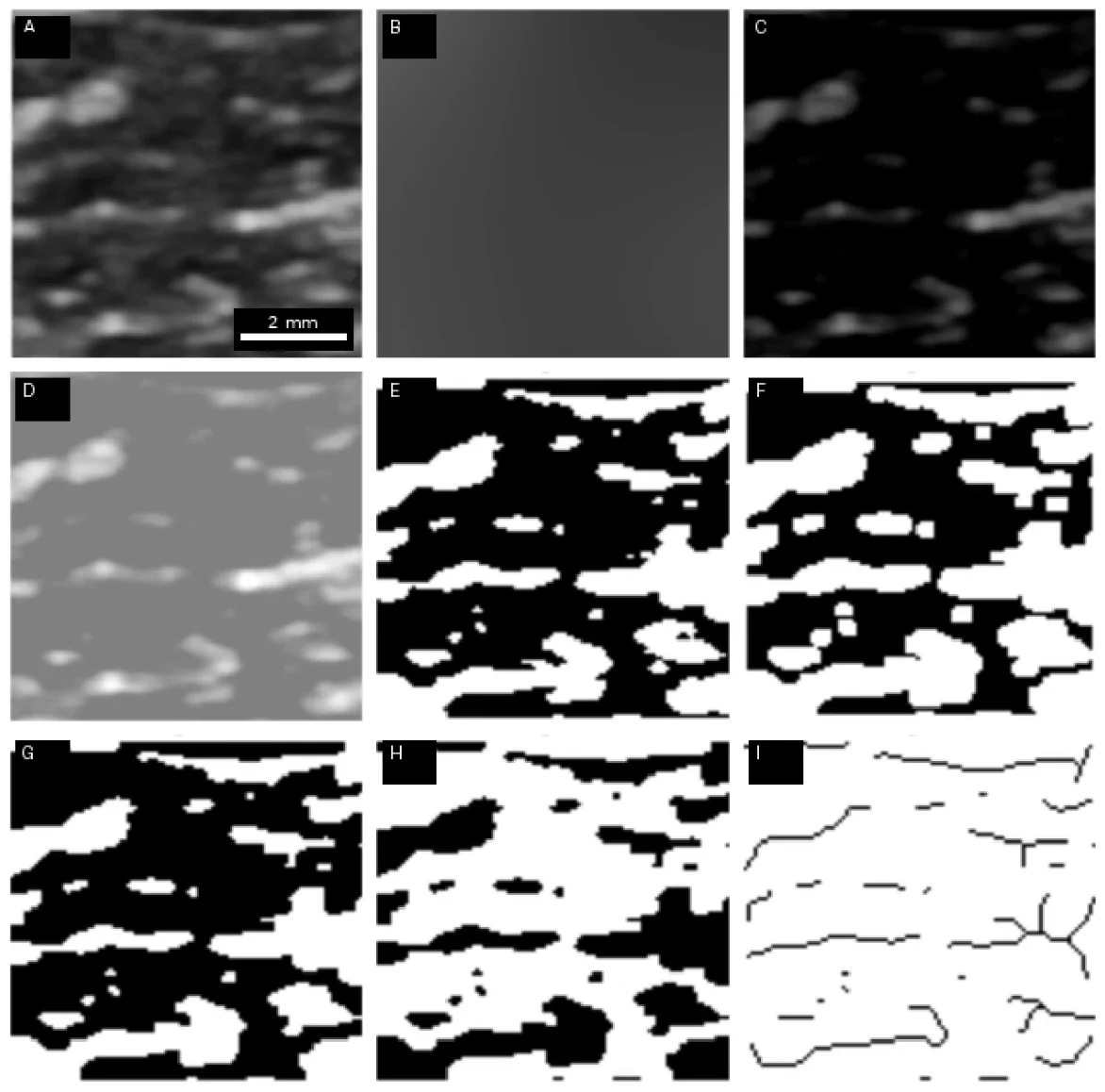
Fractal analysis steps applied to the region of interest (ROI): cropped original ROI after 8-bit conversion, before any further processing (**A**); blurred image (**B**), difference image (**C**), gray value addition (**D**), binarization (**E**), erosion (**F**), dilation (**G**), inversion (**H**), skeletonization (**I**). All panels are shown at the same scale; each panel corresponds to the 100 × 100-pixel ROI (approximately 6.8 × 6.8 mm). Scale bar: 2 mm. In the binary images, black pixels are foreground; the black skeleton pixels in I are those counted by box counting. The ROI shown is the middle ROI of the resting image in Figure 2; applying the macro to this ROI yields a box-counting FD of 1.220.

The following processing pipeline was applied to each ROI using ImageJ version 1.54f (NIH, Bethesda, USA): (1) the image was converted to 8-bit grayscale; (2) the ROI was cropped; (3) a duplicate image was created and a Gaussian blur with sigma = 35 pixels was applied; (4) the blurred image was subtracted from the original using ImageJ’s image calculator; because both images were 8-bit and no 32-bit result was requested, negative values were clipped to zero during the subtraction itself; (5) a constant value of 128 was added to each pixel to shift the histogram; (6) the image was converted to 8-bit and binarized using a fixed threshold of 0–128 (the setThreshold function in ImageJ; BlackBackground option set to false); (7) one erosion and one dilation step were applied to reduce noise; (8) the binary image was inverted; (9) the image was skeletonized using ImageJ’s Skeletonize function. The fractal dimension was then calculated using ImageJ’s built-in Fractal Box Count function with box sizes of 2, 3, 4, 6, 8, 12, 16, 32, and 64 pixels. With the BlackBackground option set to false, the skeleton is represented by black foreground pixels on a white background (as shown in Figure 3I), and these foreground (skeleton) pixels were counted. The FD values of the three ROIs were averaged to yield a single FD value per image. FD was calculated on both images acquired per side and condition, and the two image-level FD values were averaged to obtain the participant-level value for each side and condition. Both resting and clenching images were processed. Right and left sides were analyzed independently; bilateral means were also computed for each participant. The complete ImageJ macro, including bit-depth, threshold, binary foreground and box-counting settings and the example output for the ROI shown in Figure 3, is provided as Supplementary Material S1.

### 2.4. Statistical Analysis

The sample size of 30 participants per group was set in advance on the basis of feasibility. Statistical power for this fixed sample size was calculated using G*Power (v3.1.9.7; *t*-tests—Means: Difference between two independent means [two groups]; Post hoc: compute achieved power). Assuming a large effect size (Cohen’s d = 0.80), a two-tailed α of 0.05, and 30 participants per group, the achieved power was 0.861. A large effect was assumed because a previous ultrasonographic study reported a large difference in masseter thickness during clenching between bruxist and non-bruxist individuals [33]. This calculation applies to the primary outcome (masseter thickness); the FD analyses were not powered and are considered exploratory. Analyses were run in IBM SPSS 20.0 (IBM Corp., Armonk, NY, USA). The primary outcome was bilateral mean masseter muscle thickness (at rest and during clenching). FD analyses were pre-specified as exploratory secondary outcomes. Within the FD analyses, bilateral participant-level means (rest and clenching) were designated as the main comparisons, and side-specific comparisons were treated as secondary. Reported *p* values are unadjusted; as a sensitivity analysis, Holm correction was applied to the two bilateral FD comparisons and, separately, to all six FD comparisons (bilateral and side-specific). A further sensitivity analysis repeated the resting FD comparisons after excluding the two participants whose resting ROIs extended beyond the muscle. Shapiro-Wilk tested distributional normality; independent-samples *t*-test served for normally distributed variables and Mann-Whitney U for non-normal ones. Effect sizes are reported as Cohen’s d for parametric comparisons and rank-biserial correlation (r) for Mann-Whitney U comparisons [34]. 95% confidence intervals are reported for the between-group difference: for *t*-test comparisons, the mean difference with its *t*-based 95% CI; for Mann–Whitney U comparisons, the Hodges–Lehmann median difference with its distribution-free 95% CI. Chi-square compared sex distributions. Spearman correlation was used to examine the relationships between FD and muscle thickness, and between age and each measurement, within each group. The significance threshold was *p* < 0.05.

Intraobserver reliability of thickness measurement was assessed using the two measurements obtained from the two separate images acquired per side and condition (one measurement per image), across all 60 participants, with ICC calculated separately for each side and condition (60 paired measurements per estimate). ICC was calculated between the two measurements using a two-way mixed-effects model for absolute agreement and single measurements (ICC(A,1)), following Koo and Li [35]. ICC was 0.967 (95% CI: 0.95–0.98) for left resting, 0.972 (95% CI: 0.95–0.98) for left clenching, 0.980 (95% CI: 0.97–0.99) for right resting, and 0.970 (95% CI: 0.95–0.98) for right clenching measurements. FD intraobserver reliability was assessed in a randomly selected subset of 10 participants (6 with bruxism, 4 controls) by repeating the full analysis (ROI placement and processing on both images per side and condition) on the same stored images, approximately four weeks after the original analysis, with the observer blinded to the original results; FD values were averaged across the three ROIs of each image and across the two images per side and condition. ICC(A,1) was calculated separately for each side and condition, giving 10 paired observations per estimate: 0.862 (95% CI: 0.55–0.96) for left resting, 0.929 (95% CI: 0.75–0.98) for right resting, 0.850 (95% CI: 0.51–0.96) for left clenching and 0.903 (95% CI: 0.67–0.97) for right clenching. For the participant-level bilateral mean (the mean of the right and left sides), which is the unit used in the group comparisons and in the calculation of SEM and MDC95, ICC(A,1) was 0.914 (95% CI: 0.71–0.98) at rest and 0.890 (95% CI: 0.57–0.97) during clenching. The standard error of measurement was calculated as SEM = SD × √(1 − ICC), where SD is the standard deviation of all 20 bilateral values of the two analyses, and the minimal detectable change as MDC95 = 1.96 × √2 × SEM. SEM was 0.008 at rest and 0.009 during clenching, and MDC95 was 0.022 and 0.024, respectively. The MDC95 describes the smallest change in an individual participant’s bilateral FD that can be distinguished from measurement error; it is not a threshold for the statistical significance of differences between group means.

## 3. Results

The bruxism group comprised 30 patients (15 female, 15 male) with a mean age of 28.9 ± 8.7 years (range 18–47); the control group had 30 participants (15 female, 15 male) aged 29.5 ± 8.2 years on average (range 18–46). Neither age (*p* = 0.539) nor sex (*p* = 1.000) differed between groups (Table 1).

**Table 1:** Demographic Data of the Groups.

Variable	Bruxism (*n* = 30)	Healthy (*n* = 30)	*p*
Age (years)			
Mean ± SD	28.9 ± 8.7	29.5 ± 8.2	0.539
Median (Min-Max)	27.5 (18-47)	25.5 (18-46)	
Sex			
Female, *n* (%)	15 (50.0%)	15 (50.0%)	1.000
Male, *n* (%)	15 (50.0%)	15 (50.0%)	

Mann-Whitney U test was used for age comparison; Chi-square test for sex comparison. SD: Standard Deviation.

Left-side FD at rest was 1.181 ± 0.032 in bruxism patients and 1.166 ± 0.031 in controls; the difference did not reach significance (*p* = 0.068). During clenching, the left-side FD was significantly higher in the bruxism group (1.201 ± 0.033 vs. 1.178 ± 0.029; *p* = 0.006). Right-side comparisons yielded *p* = 0.078 at rest and *p* = 0.055 during clenching—neither significant. When bilateral means were used, both the resting (1.188 ± 0.026 vs. 1.171 ± 0.031; *p* = 0.029) and clenching (1.202 ± 0.027 vs. 1.182 ± 0.028; *p* = 0.008) FD values were significantly elevated in bruxism patients (Table 2). In the multiplicity sensitivity analysis, both bilateral FD differences remained significant after Holm correction across the two main comparisons (rest: *p* = 0.029; clenching: *p* = 0.016). When Holm correction was applied to all six FD comparisons, the clenching differences remained significant (left side: *p* = 0.036; bilateral: *p* = 0.041), whereas the bilateral resting difference did not (*p* = 0.114). All six muscle thickness comparisons remained significant after Holm correction (all adjusted *p* < 0.001). In two control participants, the resting muscle was thinner than the ROI height (6.8 mm), so the ROIs necessarily included adjacent non-muscle tissue. Because the analysis unit was the bilateral mean, both participants were excluded entirely from the resting comparison, including the unaffected left side of one of them. When these two participants were excluded from the resting comparison (bruxism n = 30, controls n = 28), the bilateral resting FD difference was attenuated (1.188 ± 0.026 vs. 1.174 ± 0.029; difference 0.014, 95% CI −0.001 to 0.028; independent-samples *t*-test, *t*(56) = 1.85, *p* = 0.069; Cohen’s d = 0.49). The clenching comparisons were not re-analysed because all clenching ROIs lay entirely within the muscle; all 60 participants were retained in these comparisons.

**Table 2:** Comparison of Fractal Dimension (FD) Values Between Groups.

Measurement	Condition	Bruxism (*n* = 30)		Healthy (*n* = 30)		*p*	Effect Size	Difference (95% CI)
		Mean ± SD	Median	Mean ± SD	Median			
Left Side	At Rest	1.181 ± 0.032	1.178	1.166 ± 0.031	1.158	0.068	r = 0.28	0.0151 (−0.0014, 0.0299)
	Contraction	1.201 ± 0.033	1.207	1.178 ± 0.029	1.178	0.006 **	d = 0.74	0.0228 (0.0068, 0.0388)
Right Side	At Rest	1.194 ± 0.033	1.190	1.176 ± 0.042	1.181	0.078	d = 0.46	0.0175 (−0.0020, 0.0371)
	Contraction	1.202 ± 0.028	1.204	1.186 ± 0.037	1.188	0.055	d = 0.51	0.0166 (−0.0004, 0.0336)
Mean (Right + Left)	At Rest	1.188 ± 0.026	1.186	1.171 ± 0.031	1.176	0.029 *	d = 0.58	0.0167 (0.0018, 0.0316)
	Contraction	1.202 ± 0.027	1.201	1.182 ± 0.028	1.183	0.008 **	d = 0.71	0.0197 (0.0053, 0.0341)

* *p* < 0.05; ** *p* < 0.01. Left Side Rest: Mann-Whitney U; Left Side Contraction: Independent samples *t*-test; Right Side Rest: Independent samples *t*-test; Right Side Contraction: Independent samples *t*-test; Mean (R + L) Rest: Independent samples *t*-test; Mean (R + L) Contraction: Independent samples *t*-test. SD: Standard Deviation. Effect size: Cohen’s d for *t*-test rows and rank-biserial correlation (r) for Mann–Whitney U rows; positive values indicate higher values in the bruxism group. Difference (95% CI): Bruxism − Healthy, given as the mean difference with its 95% CI from the independent-samples *t*-test *(t*-test rows) or as the Hodges–Lehmann median difference with a distribution-free 95% CI (Mann–Whitney U rows). *p* values are unadjusted.

Muscle thickness was significantly greater in bruxism patients on both sides and under both conditions (all *p* < 0.001). Left-side resting values were 12.72 ± 1.53 mm (bruxism) and 9.87 ± 1.89 mm (controls); left-side clenching values were 15.34 ± 2.03 mm and 12.47 ± 2.05 mm. Right-side figures: 13.18 ± 1.60 mm vs. 9.57 ± 1.78 mm at rest and 15.56 ± 1.96 mm vs. 12.15 ± 2.25 mm during clenching. Bilateral means showed the same pattern (rest: 12.95 ± 1.47 mm vs. 9.72 ± 1.78 mm; clenching: 15.45 ± 1.89 mm vs. 12.31 ± 2.09 mm; *p* < 0.001) (Table 3).

Within the bruxism group, FD did not differ between female and male patients (all *p* > 0.05). Clenching-phase muscle thickness was the single exception: men measured 16.24 ± 1.96 mm against women’s 14.67 ± 1.49 mm (*p* = 0.018) (Table 4).

Among controls, FD showed no sex effect (*p* > 0.05). Muscle thickness was significantly larger in men at rest (10.98 ± 1.17 mm vs. 8.46 ± 1.35 mm) and during clenching (13.96 ± 1.30 mm vs. 10.66 ± 1.22 mm), with *p* < 0.001 in both comparisons (Table 5).

**Figure 4:**
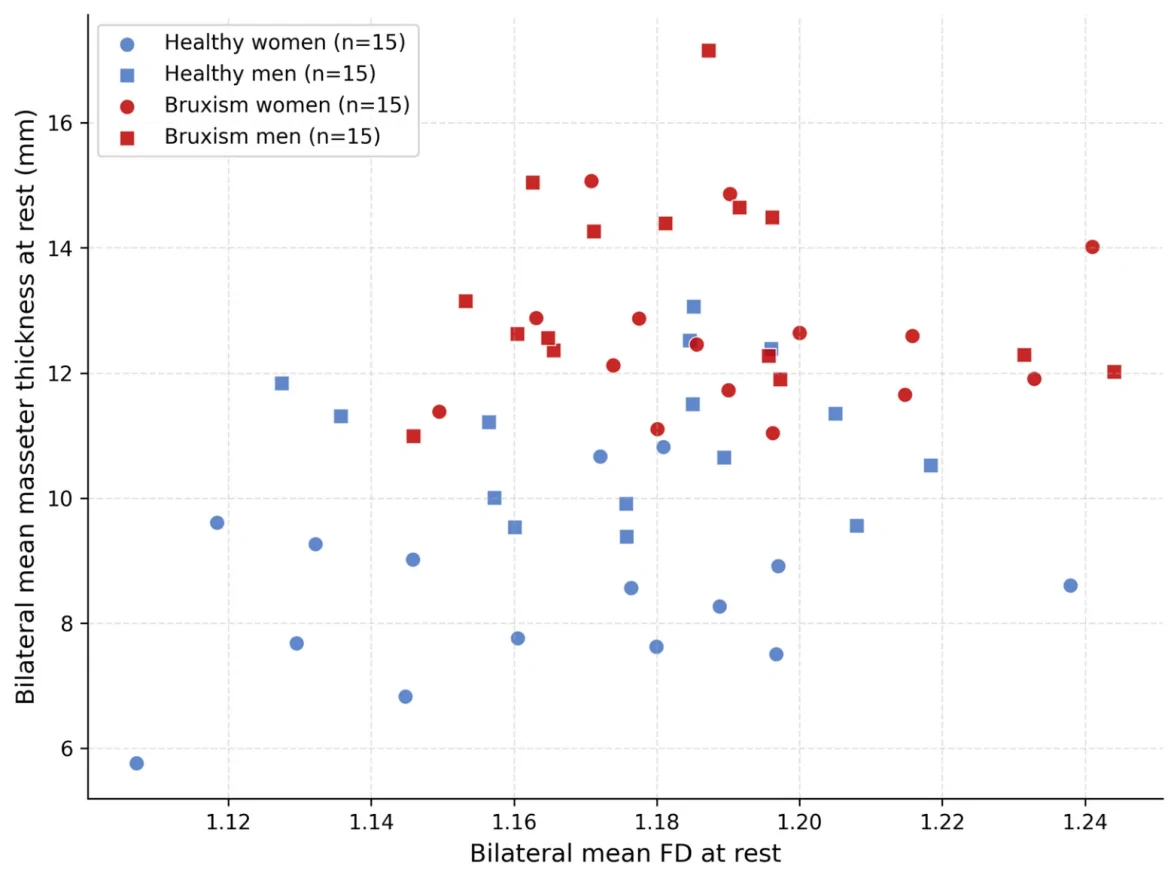
Scatter plot of bilateral mean fractal dimension (FD) versus bilateral mean masseter muscle thickness at rest. Each point represents one participant (*n* = 60); the plotted values are the same participant-level values summarized in Table 2 and Table 3. Bruxism patients are shown in red and healthy controls in blue; women are represented by circles and men by squares. Muscle thickness was higher on average in the bruxism group, but the two groups overlapped in both muscle thickness and FD.

Spearman correlations between bilateral mean FD and bilateral mean muscle thickness were weak and non-significant in both groups, at rest (bruxism: ρ = −0.11, 95% CI −0.45 to 0.26; controls: ρ = 0.17, 95% CI −0.21 to 0.50) and during clenching (bruxism: ρ = −0.30, 95% CI −0.60 to 0.08; controls: ρ = −0.04, 95% CI −0.40 to 0.32) (Table 6). The confidence intervals were wide and compatible with both negligible and moderate associations. Figure 4 presents a scatter plot of bilateral mean FD versus bilateral mean muscle thickness at rest, with the four subgroups (healthy women, healthy men, women with bruxism, men with bruxism) distinguished by different colors and symbols.

## 4. Discussion

Both muscle thickness and FD values from B-mode masseter ultrasonograms were higher in bruxism patients than in healthy controls, though the pattern of significance differed between the two parameters.

Thickness exceeded control values across all conditions—resting and clenching, right and left (Table 3). The bilateral between-group difference was similar at rest (3.23 mm; 12.95 ± 1.47 mm vs. 9.72 ± 1.78 mm) and during clenching (3.14 mm; 15.45 ± 1.89 mm vs. 12.31 ± 2.09 mm). Masseter hypertrophy related to chronic parafunctional loading is a possible explanation for this difference; however, because the exact duration of bruxism, objective loading, body mass index, and facial morphology were not systematically recorded, a causal relationship cannot be established from these cross-sectional data.

Najm’s earlier report of significantly greater clenching-phase thickness in bruxist individuals [33] is replicated here (Table 3). Arslan and Yaşar reached the same conclusion in an all-female sample [36], and Lee et al. showed that bruxism status, independent of TMJ pathology, predicts muscle bulk [37]. These findings are consistent with the long-recognized consequence of masticatory muscle hyperactivity: hypertrophy [38]. Atlihan and Bayrak similarly reported significant ultrasonographic differences in masseter muscle characteristics in patients with TMJ internal derangement [26], further supporting the utility of B-mode ultrasonography in detecting muscle-level changes associated with parafunctional loading.

When bilateral means were used, FD was significantly higher in bruxism patients at both rest (*p* = 0.029) and clenching (*p* = 0.008) (Table 2). Side-specific comparisons reached significance only for the left side during clenching (Table 2). The bilateral mean FD differences were 0.017 at rest and 0.020 during clenching. The two conditions, however, differed in robustness. The clenching difference remained significant after Holm correction across all six FD comparisons and was not affected by the ROI-related exclusion. The resting difference did not remain significant after Holm correction across six comparisons (*p* = 0.114) or after exclusion of the two control participants whose resting ROIs extended beyond the muscle (*p* = 0.069). This does not show that there is no difference at rest, but it indicates that the resting result depends on analytical choices and on the inclusion of measurements affected by non-muscle tissue. The FD findings should therefore be regarded as preliminary and exploratory, and the resting FD finding in particular should not be interpreted as an established difference.

The box-counting algorithm quantifies the complexity and heterogeneity of the image texture within the analyzed ROI. FD should be understood as a quantitative image-texture descriptor derived from the processed ultrasound image, not as a direct measure of muscle histology [27,39]. The elevated FD values observed in the bruxism group may reflect differences in image texture associated with chronic parafunctional loading, but the nature of the underlying tissue changes cannot be determined from this cross-sectional study.

Tekin et al. found that B-mode morphology, elastography, and resting FD each captured a distinct phenotypic dimension in TMJ/bruxism patients [27]. Öner Talmaç and Balkanlıoğlu observed that botulinum-toxin injection reduced masseter bulk but left FD unchanged over 90 days [28]; in the present study, FD–thickness correlations were weak and imprecisely estimated (Table 6). Polat Balkan et al. reported elevated mandibular-angle FD values on panoramic films in bruxism patients [29], suggesting that parafunctional loading may alter measurable fractal properties in the masticatory system; however, bone and muscle represent different tissues and imaging contexts. Building on these studies, the present study compared clinically assessed bruxism patients with healthy controls, analysed FD bilaterally under both resting and clenching conditions, and reported the intraobserver reliability of the FD measurement.

No statistically significant association between FD and sex was detected in this sample (Table 4 and Table 5). With 15 participants per subgroup, however, the study was not adequately powered to conclude sex independence, and this interpretation should be treated with caution. Muscle thickness was significantly greater in males in both groups (*p* < 0.001 at both conditions in controls; *p* = 0.018 during clenching in the bruxism group) (Table 4 and Table 5), consistent with published reference data [32,40].

No statistically significant association between age and any measurement was detected in this sample (Spearman ρ = −0.26 to 0.23; all *p* ≥ 0.159). This does not exclude an age effect; the relatively young and narrow-age cohort (mean age approximately 29 years in both groups) limits the ability to detect gradual age-related changes. Wider age ranges would be needed to address this question.

### Limitations

Bruxism was diagnosed clinically using structured criteria consistent with the STAB framework [15,16], rather than by polysomnography—the standard for definitive sleep bruxism confirmation. Although sleep and awake bruxism were queried during the clinical interview, the subtype was not systematically recorded, so subtype-specific analyses were not possible. Statistical power was calculated for the primary outcome (muscle thickness) at the fixed sample size (achieved power 0.861 for d = 0.80, α = 0.05, n = 30 per group); no separate calculation was conducted for FD outcomes, as reference values were unavailable at the time of study design; the FD findings should therefore be regarded as hypothesis-generating. Maximum voluntary clenching force was not objectively measured and may vary between participants. Body mass index, facial morphology, chewing habits, and prior treatment history were not systematically recorded, representing potential sources of residual confounding. Beyond the minimum one-year history required for inclusion, the exact duration of bruxism was not recorded, which limits the ability to examine associations between disease duration and ultrasonographic findings. The bilateral FD differences (0.017 at rest, 0.020 during clenching) are relatively small. They are smaller than the minimal detectable change for an individual participant (MDC95 = 0.022–0.024); this does not invalidate the group comparisons, but it indicates that small FD changes within individual patients could not be reliably detected, and neither this threshold nor the significance levels establish clinical relevance. Intraobserver reliability was excellent for muscle thickness (ICC = 0.967–0.980) and good for FD (ICC = 0.850–0.929); however, FD reliability was assessed in a subset of 10 participants by re-analysing stored images, so acquisition-related variability in FD was not evaluated. In addition, in two control participants the resting muscle was thinner than the ROI height (6.8 mm), so their resting ROIs included non-muscle tissue, and the resting FD difference did not remain significant when these participants were excluded; the resting FD findings should therefore be interpreted with particular caution. Control participants were required to have none of the four diagnostic findings, creating a particularly symptom-free comparison group; the observed differences may therefore not generalize to the more challenging clinical distinction between bruxism and other conditions presenting with tooth wear, muscle tenderness, or jaw pain.

## 5. Conclusions

Masseter muscle thickness and FD values from B-mode ultrasonograms were both higher in bruxism patients than in healthy controls, though they behaved differently across conditions and subgroups.

Thickness was significantly elevated bilaterally at rest and during clenching (all *p* < 0.001). This finding is consistent with prior literature; masseter hypertrophy related to chronic parafunctional loading is a possible, but unconfirmed, explanation.

Bilateral mean FD was modestly higher in bruxism patients during clenching. The resting FD difference was not robust to broader multiplicity correction or to exclusion of participants whose ROIs extended beyond the muscle boundaries. Further studies are needed to establish the reproducibility and clinical relevance of these texture measures.

No statistically significant association between FD and sex was detected in this sample, whereas muscle thickness was significantly greater in males. These findings are preliminary given the subgroup sizes of 15 participants each.

FD and muscle thickness showed weak, non-significant correlations within each group. Larger prospective studies incorporating polysomnographic confirmation, objective force measurement, and longitudinal design are needed to establish the clinical utility of ultrasonographic FD in bruxism assessment.

## Figures and Tables

**Table 3:** Comparison of Masseter Muscle Thickness Between Groups (mm).

Measurement	Condition	Bruxism (*n* = 30)		Healthy (*n* = 30)		*p*	Effect Size	Difference (95% CI) (mm)
		Mean ± SD	Median	Mean ± SD	Median			
Left Side	At Rest	12.72 ± 1.53	12.35	9.87 ± 1.89	9.73	<0.001 ***	r = 0.76	2.74 (1.82, 3.61)
	Contraction	15.34 ± 2.03	14.67	12.47 ± 2.05	12.13	<0.001 ***	r = 0.66	2.82 (1.82, 3.77)
Right Side	At Rest	13.18 ± 1.60	12.86	9.57 ± 1.78	9.65	<0.001 ***	d = 2.14	3.61 (2.74, 4.48)
	Contraction	15.56 ± 1.96	15.22	12.15 ± 2.25	12.14	<0.001 ***	d = 1.62	3.41 (2.32, 4.50)
Mean (Right + Left)	At Rest	12.95 ± 1.47	12.78	9.72 ± 1.78	9.73	<0.001 ***	r = 0.85	3.14 (2.27, 4.01)
	Contraction	15.45 ± 1.89	15.10	12.31 ± 2.09	12.26	<0.001 ***	r = 0.73	3.09 (2.02, 4.16)

*** *p* < 0.001. Left Side Rest: Mann-Whitney U; Left Side Contraction: Mann-Whitney U; Right Side Rest: Independent samples *t*-test; Right Side Contraction: Independent samples *t*-test; Mean (R + L) Rest: Mann-Whitney U; Mean (R + L) Contraction: Mann-Whitney U. SD: Standard Deviation. Effect size: Cohen’s d for *t*-test rows and rank-biserial correlation (r) for Mann–Whitney U rows; positive values indicate higher values in the bruxism group. Difference (95% CI): Bruxism − Healthy, given as the mean difference with its 95% CI from the independent-samples *t*-test (*t*-test rows) or as the Hodges–Lehmann median difference with a distribution-free 95% CI (Mann–Whitney U rows). *p* values are unadjusted.

**Table 4:** Comparison of FD and Muscle Thickness by Sex in the Bruxism Group.

Variable	Female (*n* = 15)		Male (*n* = 15)		*p*	Effect Size	Difference (95% CI)
	Mean ± SD	Median	Mean ± SD	Median			
FD at Rest (Mean)	1.192 ± 0.025	1.191	1.183 ± 0.028	1.180	0.368	d = 0.33	0.0089 (−0.0110, 0.0287)
FD at Contraction (Mean)	1.208 ± 0.022	1.207	1.196 ± 0.031	1.199	0.228	d = 0.45	0.0122 (−0.0081, 0.0324)
Muscle Thickness at Rest (mm, Mean)	12.55 ± 1.25	12.53	13.34 ± 1.61	13.10	0.144	d = −0.55	−0.79 (−1.87, 0.29)
Muscle Thickness at Contraction (mm, Mean)	14.67 ± 1.49	14.54	16.24 ± 1.96	16.35	0.018 *	r = −0.51	−1.36 (−2.70, −0.44)

* *p* < 0.05. FD at Rest: Independent-samples *t*-test; FD at Contraction: Independent-samples *t*-test; Muscle Thickness at Rest: Independent-samples *t*-test; Muscle Thickness at Contraction: Mann-Whitney U. SD: Standard Deviation. Effect size: Cohen’s d for *t*-test rows and rank-biserial correlation (r) for Mann–Whitney U rows; negative values indicate higher values in males. Difference (95% CI): Female − Male, given as the mean difference with its 95% CI from the independent-samples *t*-test (*t*-test rows) or as the Hodges–Lehmann median difference with a distribution-free 95% CI (Mann–Whitney U rows). *p* values are unadjusted.

**Table 5:** Comparison of FD and Muscle Thickness by Sex in the Healthy Group.

Variable	Female (*n* = 15)		Male (*n* = 15)		*p*	Effect Size	Difference (95% CI)
	Mean ± SD	Median	Mean ± SD	Median			
FD at Rest (Mean)	1.165 ± 0.035	1.163	1.177 ± 0.026	1.181	0.265	d = −0.42	−0.0128 (−0.0359, 0.0103)
FD at Contraction (Mean)	1.183 ± 0.027	1.178	1.181 ± 0.031	1.188	0.864	d = 0.06	0.0018 (−0.0198, 0.0234)
Muscle Thickness at Rest (mm, Mean)	8.46 ± 1.35	8.29	10.98 ± 1.17	11.05	<0.001 ***	d = −2.00	−2.52 (−3.47, −1.58)
Muscle Thickness at Contraction (mm, Mean)	10.66 ± 1.22	10.53	13.96 ± 1.30	13.68	<0.001 ***	d = −2.62	−3.30 (−4.25, −2.36)

*** *p* < 0.001. All comparisons: Independent samples *t*-test. SD: Standard Deviation. Effect size: Cohen’s d for *t*-test rows and rank-biserial correlation (r) for Mann–Whitney U rows; negative values indicate higher values in males. Difference (95% CI): Female − Male, given as the mean difference with its 95% CI from the independent-samples *t*-test (*t*-test rows) or as the Hodges–Lehmann median difference with a distribution-free 95% CI (Mann–Whitney U rows). *p* values are unadjusted.

**Table 6:** Correlation Between FD and Masseter Muscle Thickness Within Each Group.

Variable	Spearman ρ (95% CI)	*p*
FD vs. Muscle Thickness at Rest (Bruxism)	−0.11 (−0.45, 0.26)	0.569
FD vs. Muscle Thickness at Contraction (Bruxism)	−0.30 (−0.60, 0.08)	0.107
FD vs. Muscle Thickness at Rest (Healthy)	0.17 (−0.21, 0.50)	0.370
FD vs. Muscle Thickness at Contraction (Healthy)	−0.04 (−0.40, 0.32)	0.827

Spearman correlation coefficient was used. ρ = Spearman’s rho. *p* values are unadjusted. 95% CIs were obtained by Fisher z-transformation using the Bonett–Wright standard error.

## Data Availability

The data presented in this study are available on request from the corresponding author.

## Supplementary Materials

The following supporting information can be downloaded at: https://www.mdpi.com/article/10.3390/diagnostics16213648/s1, Material S1: ImageJ macro and settings used for fractal dimension analysis, with example output.
