# Supplementary material for: Ultrasonographic Evaluation of Masseter Muscle Thickness and Fractal Dimension in Bruxist and Healthy Individuals
Source: Diagnostics (Basel). 2026 Nov 4;16(21):3648. doi: 10.3390/diagnostics16213648 (PMC13763388; doi:10.3390/diagnostics16213648)
Supplement: Supplementary file 1 [file diagnostics-16-03648-s001.zip › diagnostics-4634213-supplementary.pdf]

Material S1: ImageJ macro and settings used for fractal dimension analysis, with example output

Ultrasonographic Evaluation of Masseter Muscle Thickness and Fractal Dimension in Bruxist and Healthy Individuals

The following macro was applied, without modification, to each 100 × 100-pixel region of interest (ROI) in ImageJ version 1.54f (NIH, Bethesda, MD, USA). Before running the macro, the ROI was selected on the 8-bit image (Edit → Selection → Specify, width = 100, height = 100 pixels).

Macro

```
run("8-bit");
run("Crop");
run("Duplicate...", "title=f");
run("Duplicate...", "title=f1");
selectWindow("f1");
run("Gaussian Blur...", "sigma=35");
imageCalculator("Subtract create", "f","f1");
selectWindow("Result of f");
run("Add...", "value=128");
run("8-bit");
setAutoThreshold("Default");
setThreshold(0, 128);
setOption("BlackBackground", false);
run("Convert to Mask");
selectWindow("Result of f");
run("Erode");
selectWindow("Result of f");
run("Dilate");
selectWindow("Result of f");
run("Invert");
selectWindow("Result of f");
run("Skeletonize");
selectWindow("Result of f");
run("Fractal Box Count...", "box=2,3,4,6,8,12,16,32,64");
```

Settings and conventions

- Bit depth: 8-bit throughout. The subtraction is performed on 8-bit images without a 32-bit result, so negative differences are set to zero before the constant of 128 is added.
- Gaussian blur: sigma = 35 pixels.
- Threshold: fixed at 0–128 (setThreshold); the preceding setAutoThreshold("Default") call only initialises the threshold display. Because the threshold lies at the added constant (128), pixels are classified by the sign of the difference between the original and the blurred image; the binary image is therefore identical whether or not negative differences are clipped.
- Binary convention: BlackBackground = false, so foreground pixels are black on a white background.
- Box counting: ImageJ Fractal Box Count with box sizes of 2, 3, 4, 6, 8, 12, 16, 32 and 64 pixels; the counted pixels are the black (foreground) skeleton pixels shown in Figure 3I. FD is the negative slope of the regression of log(count) on log(box size).

Example output (ROI shown in Figure 3)

Applying the macro to the ROI shown in Figure 3 (middle ROI of the resting image in Figure 2) gives the following box counts:

Box size (pixels)	2	3	4	6	8	12	16	32	64
Count	308	213	174	124	95	64	43	14	4

Fractal dimension (D) = 1.220.

The original ImageJ output for this ROI (Results table of the Fractal Box Count function) and the corresponding log–log plot are shown below.

Results

	Label	C2	C3	C4	C6	C8	C12	C16	C32	C64	D
1	Result	308	213	174	124	95	64	43	14	4	1.220

ImageJ Results table for the ROI shown in Figure 3: box counts (C2–C64) and fractal dimension (D).

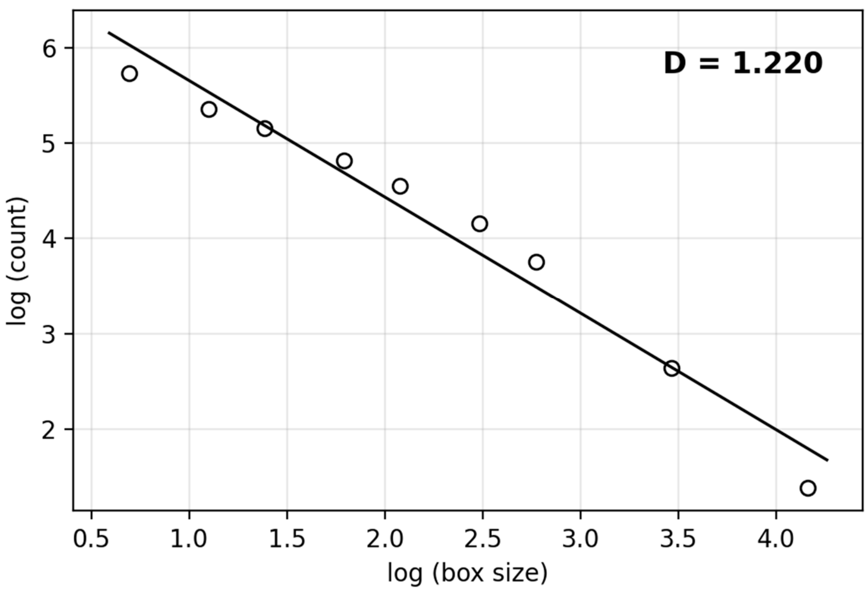

Log–log plot of the box counts above; the negative slope of the regression line gives FD = 1.220.
